# Higher Serum 25-Hydroxyvitamin D Concentrations Associate with a Faster Recovery of Skeletal Muscle Strength after Muscular Injury

**DOI:** 10.3390/nu5041253

**Published:** 2013-04-17

**Authors:** Tyler Barker, Vanessa T. Henriksen, Thomas B. Martins, Harry R. Hill, Carl R. Kjeldsberg, Erik D. Schneider, Brian M. Dixon, Lindell K. Weaver

**Affiliations:** 1 The Orthopedic Specialty Hospital, Murray, UT 84107, USA; E-Mail: vanessa.henriksen@imail.org; 2 ARUP Laboratories, Institute for Clinical and Experimental Pathology, Salt Lake City, UT 84108, USA; E-Mails: martintb@aruplab.com (T.B.M.); harry.hill@path.utah.edu (H.R.H.); ckbike@comcast.net (C.R.K.); 3 Department of Pathology, Pediatrics and Medicine, University of Utah, Salt Lake City, UT 84132, USA; 4 USANA Health Sciences, Inc., Salt Lake City, UT 84120, USA; E-Mails: erik.schneider@us.usana.com (E.D.S.); brian.dixon@us.usana.com (B.M.D.); 5 Hyperbaric Medicine, Intermountain Medical Center, Murray, UT 84107, USA; E-Mail: lindell.weaver@imail.org; 6 LDS Hospital, Salt Lake City, UT 84143, USA; 7 School of Medicine, University of Utah, Salt Lake City, UT 84132, USA

**Keywords:** vitamin D, exercise, muscle strength, cytokines, albumin

## Abstract

The primary purpose of this study was to identify if serum 25-hydroxyvitamin D (25(OH)D) concentrations predict muscular weakness after intense exercise. We hypothesized that pre-exercise serum 25(OH)D concentrations inversely predict exercise-induced muscular weakness. Fourteen recreationally active adults participated in this study. Each subject had one leg randomly assigned as a control. The other leg performed an intense exercise protocol. Single-leg peak isometric force and blood 25(OH)D, aspartate and alanine aminotransferases, albumin, interferon (IFN)-γ, and interleukin-4 were measured prior to and following intense exercise. Following exercise, serum 25(OH)D concentrations increased (*p* < 0.05) immediately, but within minutes, subsequently decreased (*p* < 0.05). Circulating albumin increases predicted (*p* < 0.005) serum 25(OH)D increases, while IFN-γ increases predicted (*p* < 0.001) serum 25(OH)D decreases. Muscular weakness persisted within the exercise leg (*p* < 0.05) and compared to the control leg (*p* < 0.05) after the exercise protocol. Serum 25(OH)D concentrations inversely predicted (*p* < 0.05) muscular weakness (*i.e.*, control leg *vs.* exercise leg peak isometric force) immediately and days (*i.e.*, 48-h and 72-h) after exercise, suggesting the attenuation of exercise-induced muscular weakness with increasing serum 25(OH)D prior to exercise. Based on these data, we conclude that pre-exercise serum 25(OH)D concentrations could influence the recovery of skeletal muscle strength after an acute bout of intense exercise.

## 1. Introduction

Vitamin D is a pleiotropic micronutrient that influences health across a range of physiological and pathophysiological conditions in humans. In the body, 25-hydroxyvitamin D (25(OH)D) is the most abundant circulating metabolite and most reliable indicator of vitamin D intake and storage [1]. Maintaining an adequate circulating concentration of 25(OH)D is, therefore, important to the physiological events moderated by vitamin D.

In addition to its well-known regulation of calcium and mineral homeostasis, vitamin D also regulates genomic and non-genomic events that govern skeletal muscle function, for review see, for review [2,3,4,5,6]. These functions include the regulation of calcium handling and transport, the expression of cytoskeletal proteins, phosphate metabolism, cell proliferation and differentiation, and the activation of mitogen activated protein kinase signaling pathways in skeletal muscle [7,8,9,10,11,12,13,14,15,16,17,18,19,20,21,22]. These functions are established in experimental animal and cell culture studies, but less clear is the regulatory influence of vitamin D on skeletal muscle function in humans. 

In physiological and pathophysiological conditions, muscular strength associates with serum 25(OH)D concentrations [23,24,25,26,27,28,29,30,31,32,33,34,35]. This association is prominent at deficient (<20 ng/mL) and insufficient (20 to 32 ng/mL) serum 25(OH)D concentrations [25,36,37]. Despite the efficacy of supplemental vitamin D to increase serum 25(OH)D concentrations, however, ambiguity persists regarding the influence of supplemental vitamin D on skeletal muscle strength [30,38,39,40,41,42,43]. Nevertheless, it is inferred that increasing serum 25(OH)D from deficient to insufficient or sufficient (>32 ng/mL) concentrations attenuates muscular weakness. 

Immediate (*i.e.*, minutes to hours) and persistent (*i.e.*, days) muscular weakness can follow intense exercise or a muscle-damaging event [44,45]. Muscular weakness after intense exercise or a muscle-damaging event could cause a predisposition to additional trauma. For example, muscular weakness due to injury could contribute to the development of stress fractures in military recruits, which interestingly, could be prevented by increasing serum 25(OH)D concentrations [46,47]. Likewise, low serum 25(OH)D concentrations contribute to muscular weakness and the increase in falls and bone fractures in elderly [36,48,49,50,51,52,53,54]. Increasing or maintaining an adequate serum 25(OH)D concentration, therefore, could preserve muscular strength after injury and protect against further injury.

Following intense exercise, serum 25(OH)D concentrations were not associated with muscular strength [55]. In that previous study, however, the serum 25(OH)D concentration and muscular strength relationship was examined 2-days after eccentric contractions [55]. In experimental rats, vitamin D (activated 7-dehyrocholesterol at 332,000 IU/kg of body mass) promoted muscle regeneration and accelerated the recovery of skeletal muscle strength after crush injury [19], which could possibly relate to the ability of vitamin D to regulate satellite cells in the repair of skeletal muscle [21].

Eccentric (*i.e.*, active-lengthening) contractions of skeletal muscle induce muscle injury and a systemic inflammatory response [56], which in theory, could contribute to serum 25(OH)D concentration decreases [57,58,59]. Although the causative mechanism responsible for the serum 25(OH)D decrease during inflammation awaits future elucidation in humans, persuasive results reveal the regulatory influence of inflammatory cytokines on vitamin D metabolism in immune cells. 

Interferon (IFN)-γ is an inflammatory cytokine that influences vitamin D metabolism in immune cells [60,61]. In isolated peripheral immune cells, IFN-γ increases 1α-hydroxylase activity and mediates the conversion of 25(OH)D to 1,25-dihydroxyvitamin D (1,25(OH)D) [60,61,62,63,64,65]. In contrast to IFN-γ, interleukin (IL)-4 is a cytokine that initiates the catabolism of 25(OH)D to 24,25-dihydroxyvitamin D [65]. Following intense exercise, IFN-γ decreases or remains unchanged [66,67,68] while IL-4 remains unchanged or below detection limits in the circulation [69,70]. Thus, given its regulation of vitamin D metabolism in immune cells, IFN-γ changes could be inversely related to serum 25(OH)D concentration alterations after exercise. 

Data suggest that serum 25(OH)D concentrations and muscular strength are not associated after exercise [55]. However, in that earlier study [55], it is possible that the imposed exercise protocol induced a systemic inflammatory response [56]. This is an important consideration because inflammation could modulate serum 25(OH)D concentrations, and consequentially, confound the interpretation between vitamin D and muscle strength [71]. To address this conundrum, the primary aim of this study was to identify if pre-exercise serum 25(OH)D concentrations predict immediate and persistent (*i.e.*, several days) muscular weakness after intense exercise. We hypothesized that pre-exercise serum 25(OH)D concentrations inversely predict immediate and persistent muscular weakness after intense exercise. As the secondary aim of this study, we sought to identify if circulating cytokines predict serum 25(OH)D concentrations after intense exercise. We hypothesized that IFN-γ changes inversely predict serum 25(OH)D concentration alterations.

## 2. Experimental Section

The Institutional Review Board at Intermountain Healthcare (Murray, UT, USA) approved this study. Recreationally active (*i.e.*, at least 30 min of continuous exercise 3 times per week for 1 year prior to enrollment) subjects were informed of the experimental protocol and procedures and provided both written and verbal consent prior to participation. Subjects were excluded if: they were taking any dietary supplements or anti-inflammatory medications, suffered a lower leg injury during the previous year that required the use of crutches, were taking digoxin and antiarrhythmic medication(s), diagnosed with diabetes mellitus, impaired liver or kidney function, pregnant, morbidly obese (body mass index (BMI) > 40 kg/m^2^), using corticosteroid medication, planning on increasing or decreasing the amount of time spent in the sun or tanning bed, or traveling south of 37°N in latitude during study participation. Subjects were also excluded if they had a history of: metabolic bone disease, skeletal muscle pathology, cardiac or peripheral cardiovascular system abnormalities, clotting disorders, coronary artery disease, peripheral vascular disease, stroke, cancer, hypercalcemia or parathyroid dysfunction, iron deficiency within the past year or a potential concern of iron deficiency, high cholesterol or triglycerides, high blood pressure, or the presence of strength or power output asymmetry (*i.e.*, >5% difference in peak isometric between legs). Potential subjects were excluded from study participation if they reported or showed any medical condition, under physician treatment, or taking any prescribed medication. During study participation, subjects were asked to keep their diet consistent with their regular eating habits during the previous year.

Data was collected during the winter (December to March) in Salt Lake City, UT, USA (40°N latitude). Subjects were asked to refrain from physical activity, and using steroids, aspirin, ibuprofen, naproxen sodium, acetaminophen, or other anti-inflammatory agents 72-h prior to the blood draw and strength testing procedures to remove the potential confounding influences of activity and anti-inflammatory agents on blood and strength testing results.

### 2.1. Exercise Protocol

**Figure 1:**
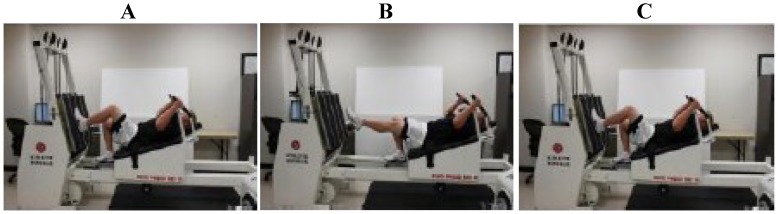
Examples of the single-leg strength testing and the intense-stretch shortening contraction (SSC) protocol on the horizontal Plyo-press. (**A**) The weight stack resistance was overloaded to prevent sled movement in order to achieve isometric testing. (**A**–**C**) Subjects performed repetitive single-leg jumps during the SSC protocol. Illustrated below is an example of a subject in the push-off position (**A**), at peak height of the jump (**B**), and the subsequent landing (**C**) during the SSC protocol. Subjects performed 10 sets of 10 jumps with a 20-s rest between each set at 75% of body mass on one leg only (*i.e.*, SSC leg). Note: This figure is adapted with permission from [68]. Copyright © Barker *et al*.; licensee BioMed Central Ltd.

In an attempt to minimize the bias of leg dominance, subjects that displayed greater than a 5% difference in peak isometric force between legs were excluded from study participation. Following study inclusion, each subject had one leg randomly assigned as the control (CON) leg. The other leg performed an intense-stretch shortening contraction (SSC) protocol (Figure 1). The rationale for performing this protocol unilaterally was to assess isometric force within and between legs across time, and to allow each subject to serve as their own control.

The purpose of the SSC protocol was to induce peak isometric force deficits that would persist for several days [68]. The SSC protocol consisted of 10 sets of 10 repetitive jumps at 75% of body mass with a 20 s rest between each set. Subjects were instructed and verbally encouraged to perform each set with maximal effort and to jump as high as possible through a full range of motion (90° of knee flexion-to-full extension). Subjects performed presses through a full range of motion if they were unable to complete the jumps. If they were unable to complete presses, the exercise protocol was terminated. The mean number of jumps and presses completed during the SSC protocol were 62 ± 9 and 23 ± 5, respectively, while 7 (3 males and 4 females; age, 32 ± 3 years; height, 167 ± 5 cm; body mass, 76.8 ± 8.7 kg; body mass index, 27.1 ± 1.7 kg/m^2^) of the 14 subjects were unable to complete the SSC protocol due to fatigue or exhaustion. Of the subjects who completed the exercise protocol, 3 subjects (all males; age, 29 ± 2 years; height, 182 ± 4 cm; body mass, 85.3 ± 5.4 kg; body mass index, 25.7 ± 1.6 kg/m^2^) completed all the jumps and four subjects (3 males and 1 female; age, 33 ± 2 years; height, 176 ± 7 cm; body mass, 78.0 ± 7.5 kg; body mass index, 24.8 ± 0.8 kg/m^2^) completed the exercise protocol by performing a combination of jumps and presses. Circulating chemistries and leg strength were not significantly different between subjects who completed the exercise protocol compared to those who were unable to complete the exercise protocol (data not shown). Although 7 subjects were unable to complete the acute exercise protocol, all subjects (*n* = 14), completed the remainder of study protocol following the exercise protocol.

**Figure 2:**
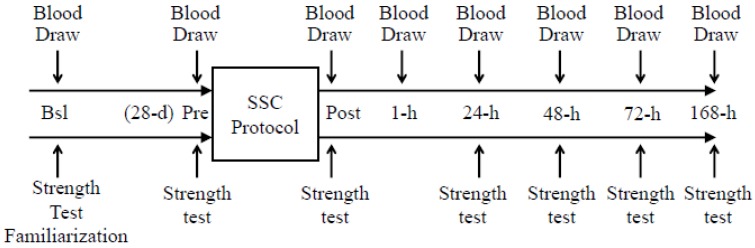
Study protocol. Each subject provided eight fasting blood draws. The first blood draw was performed at baseline (Bsl) and 28-day before the SSC protocol. The rationale for collecting this sample 28-day before the SSC protocol was to allow for the seasonal decrease in serum 25(OH)D concentrations. The second blood draw was obtained immediately before (Pre) the SSC protocol. The six remaining blood draws were performed immediately (*i.e.*, Post), 1-h, 24-h, 48-h, 72-h and 168-h (*i.e.*, 7 days) after the SSC protocol. Subjects were familiarized with the single-leg peak isometric force testing procedure at Bsl. Thereafter, single-leg peak isometric force measurements were performed on six different occasions: (1) immediately before (Pre) the SSC protocol; and (2) immediately (Post); (3) 24-h; (4) 48-h; (5) 72-h and (6) 168-h after the SSC protocol.

Subjects were allowed to consume water *ad libitum* during and following exercise protocol. The study protocol is illustrated in Figure 2.

### 2.2. Analytical Procedures

Blood draws were performed from the antecubital vein and collected in one 6.0 mL red-top serum Becton Dickinson (BD; Franklin Lake, NJ, USA) vacutainer tube, one 4.0 mL purple-top BD Vacutainer tube (K2 EDTA 7.2 mg plasma), and one 4.5 mL light green-top BD Vacutainer tube (PST Gel and Lithium Heparin, 83 units). After coagulation, serum was separated by centrifugation (VWR International, Clinical 50 Centrifuge, Radnor, PA, USA) at 1100× *g* for 20 min at room temperature. Serum samples were immediately aliquoted to several 500 µL microcentrifuge tubes and stored at −80 °C (Revco Freezer, GC Laboratory Equipment, Asheville, NC, USA) until the day of 25(OH)D and cytokine concentration analyses (see below). Plasma was separated by centrifugation (Heraeus Labofuge 400 series, Buckinghamshire, England) at 2400× *g* for 6 min at room temperature within 20 min of sample collection. Following separation, plasma samples were sent to ARUP Laboratories (Salt Lake City, UT, USA) for parathyroid hormone (PTH), calcium, albumin, aspartate aminotransferase (AST), and alanine aminotransferase (ALT) chemistries (see below).

#### 2.2.1. Serum 25(OH)D Concentration

Serum 25(OH)D concentrations (ng/mL) were measured in duplicate (coefficient of variation = 3.19%) at USANA Health Sciences, Inc. (Salt Lake City, UT, USA) [39]. Specifically, 100 µL of serum was added to 400 µL of a 2:1 methanol:chloroform solution containing deuterated 25(OH)D_3_ as an internal standard (10 ng/mL in the stock solution; 40 ng/mL final concentration) in a 2 mL centrifuge tube. Samples were immediately vortexed and allowed to sit on ice for 10 min. Samples were then spun at 15,000× *g* for 5 min and the supernatant was transferred to a new 2 mL centrifuge tube containing 500 µL of chloroform. Samples were vortexed and allowed to stand for 5 min. To achieve phase separation, 750 µL of ddH_2_O was added, vortexed, and centrifuged for 2 min at 15,000× *g*. The aqueous phase (top), and any debris between the two phases, was removed and discarded. The remaining organic phase was dried down to completeness in a centrifugal vacuum concentrator for 18 min at 45 °C under negative pressure. The pellet was then resuspended in 100 µL of methanol and added to high performance-liquid chromatography (HPLC) vials.

Analytes were separated by injecting 10 µL into an Agilent HPLC (series 6410, Model G6410B, Santa Clara, CA, USA) and a Phenomenex Inertsil 3 micron, 150 × 4.60 mm column. Method conditions were: 0–8 min, 90% MeOH/10% (0.03% formic acid in water); 8–15 min, 100% 2-propanol; 15–20 min, 90% MeOH/10% (0.03% formic acid in water). 25(OH)D_2_ and 25(OH)D_3_ were detected on an Agilent tandem mass spectrometer (Series 6410, Model G6410B, Santa Clara, CA, USA) using atmospheric pressure chemical ionization (APCI) detection (350 °C gas temperature, 400 °C vaporizer). The 25(OH)D_3_, deuterated 25(OH)D_3_, and 25(OH)D_2_ precursor ions were 383.3, 386.3, and 395.4, respectively. The 25(OH)D_3_, deuterated 25(OH)D_3_, and 25(OH)D_2_ product ions were 365.3, 368.3, and 208.9, respectively. Serum 25(OH)D_2_ and 25(OH)D_3_ concentrations were determined relative to authentic standards and corrected for recovery of the 25(OH)D_3_ internal standard. The detection limit was <1 ng/mL for all analytes. The sum of 25(OH)D_2_ and 25(OH)D_3_ concentrations was used as the 25(OH)D total concentration. Serum 25(OH)D_2_ was not detected in any of the subjects, and therefore, serum 25(OH)D total concentrations are referred to as serum 25(OH)D concentrations hereafter. 

#### 2.2.2. Serum Cytokine Concentrations

Serum IFN-γ and IL-4 were quantitated using a multiplexed sandwich capture assay developed in the ARUP Institute for Clinical and Experimental Pathology (University of Utah, Salt Lake City, UT, USA) using the Luminex Multi-Analyte Profiling system (Luminex, Austin, TX, USA) [72,73]. Monoclonal capture antibodies for IFN-γ and IL-4 were coupled to microspheres (Luminex). The monoclonal antibody for IL-4 was purchased from Pharmingen/BD Biosciences (San Diego, CA, USA) and the monoclonal antibody for IFN-γ was purchased from Biosciences (San Diego, CA, USA). 

Standard curves for each cytokine were made using known concentrations of recombinant human cytokine of interest and performed during the same run as the subjects’ serum analyses. All incubations were conducted at room temperature on an orbital plate shaker while protected from light for 10 min. Following incubation, beads (25 μL) were added to each well after 2 repeated bouts of vortexing (10 s) and sonication (20 s). The plate was then incubated for 1 h, washed 3× by vacuum filtration with phosphate-buffered saline containing Tween 20 (PBST). Then, 100 μL of a mixture of 2 different biotinylated secondary antibodies were added and incubated for 30 min. Following washes (3×) with PBST, 100 μL 5 μg streptavidin-conjugated *R*-phycoerythrin/mL (Moss Substrates, Pasadena, MD, USA) were added to each well. Plates were incubated for 20 min, washed 3× with PBST, then beads were re-suspended in 150 μL PBST and mixed for 5 min. 

Microtiter plates were then placed in a Luminex 100 instrument for analysis. Microspheres pass through a flow cell where dual lasers identify the microsphere and quantitate the amount of analyte bound to the microsphere by measuring the median fluorescent intensity of the reporter molecule (phycyoerythrin). The median fluorescence intensity is then converted in pg/mL based on the known concentrations of the standard curve. 

#### 2.2.3. Clinical Chemistries

Plasma AST (U/L), ALT (U/L), PTH (pg/mL), and calcium (mg/dL) concentrations were measured using a quantitative electrochemiluminescent immunoassay, and plasma albumin (g/dL) concentrations were determined using spectrophotometry (ARUP Laboratories, Salt Lake City, UT, USA).

#### 2.2.4. Single-Leg Strength Testing

Single-leg strength testing was performed on a horizontal Plyo-Press (Athletic Republic, Park City, UT, USA). The reliability (intraclass reliability coefficients = 0.98) for the peak isometric force measurement on the Plyo-Press has been reported previously [68] along with the testing procedures [39,74]. In brief, the Plyo-Press sled was adjusted for each subject to align the knee and hip joint flexion angles to 90° with the abdominal, low back region secured and stabilized to the Plyo-Press sled with a harness. Hip and knee extension-isometric contractions were accomplished by overloading the weight stack resistance (>2260 N). Leg selection (*i.e.*, CON or SSC leg) at the start of each testing session was randomized and followed by an alternating sequence of leg contractions. Peak isometric contractions on each leg were performed in triplicate (CON coefficient of variation = 3.62%; SSC leg coefficient of variation = 4.70%), and each isometric contraction was 3 s in duration and separated by 1 min of rest. Subjects were verbally instructed and strongly encouraged to exert maximal force against the mounted force platform during each isometric contraction. Force output was measured from signals obtained from a mounted force plate (Advanced Mechanical Technology, Watertown, MA, USA) on the Plyo-press. Data were sampled at 200 Hz with a low-pass filter at 10 Hz using DartPower software (Athletic Republic, Park City, UT, USA, version 2.0). Peak isometric force was defined as the highest resultant force produced from the three isometric tests and was expressed relative to body mass (N/kg).

### 2.3. Statistical Analyses

Data were checked for normality prior to all statistical analyses with a Kolmogorov-Smirnov test. Statistical significance of serum 25(OH)D, cytokine, PTH, AST, and ALT concentration data were assessed with a Friedman repeated measures analysis of variance (ANOVA) on ranks followed by a Tukey’s Honestly Significant Difference (HSD) to test multiple pairwise comparisons. Statistical significance of calcium and albumin concentration data were assessed with a one-way ANOVA with repeated measures followed by a Tukey’s HSD to test multiple pairwise comparisons. Statistical significance of the muscle strength data was assessed with a two-way (time, leg) ANOVA with repeated measures and followed by a Holm-Sidak method to test for multiple pairwise comparisons. 

Separate multiple linear regression analyses were performed to determine if Pre serum 25(OH)D concentrations or the change in serum 25(OH)D concentrations from Bsl to Pre predict the immediate (*i.e.*, Post) and persistent (*i.e.*, 24-h, 48-h, and 72-h) deficits in muscle strength after a muscle-damaging event. Along with Pre serum 25(OH)D concentrations and the change in serum 25(OH)D concentrations from Bsl to Pre, body mass index and age were included as independent variables in the multiple linear regression model. Another multiple linear regression was performed to determine if circulating deviations in IFN-γ, calcium, PTH, or albumin predict serum 25(OH)D alterations. Prior to all multiple linear regressions, serum 25(OH)D, IFN-γ, and PTH concentrations were rank transformed in order to achieve normality. Selection of independent variables for the multiple linear regression models were based on their biological significance on the dependent variable. 

Secondary analyses consisted of a one (gender)-way ANOVA with repeated measures were performed to determine if a statistical difference existed between gender data (*i.e.*, serum 25(OH)D, peak isometric force, peak isometric force difference between the CON and SSC legs at Post, 24-h, 48-h, and 72-h, AST, ALT, albumin, calcium, iPTH, IFN-γ and IL-4). Statistical analyses were performed with SysStat software (SigmaPlot 10.0, SigmaStat 3.5, Chicago, IL, USA) and significance was set at a *p* < 0.05. Data presented as mean ± SEM, unless otherwise noted.

## 3. Results

### 3.1. Subject Characteristics

Fourteen non-smoking subjects (male, *n* = 9; female, *n* = 5; age, 32 ± 1 year; height, 173 ± 5 cm; body mass, 79.0 ± 4.8 kg; body mass index, 26.1 ± 0.9 kg/m^2^) participated in this study. No subjects reported being highly trained, actively competing at a professional or amateur level, or performing recreational activities on a daily basis or for extended durations (*i.e.*, >60 min per session) consistently one year prior to study enrollment.

### 3.2. Serum 25(OH)D Concentrations

**Figure 3:**
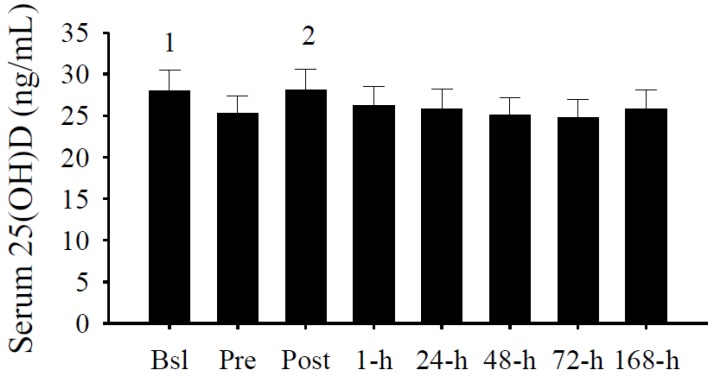
Serum 25(OH)D concentrations (ng/mL). Serum 25(OH)D concentrations were significantly increased at Bsl (^1^ *p* < 0.05 *vs.* Pre, 48-h, and 72-h) and Post (^2^ *p* < 0.05 *vs.* Pre, 24-h, 48-h, 72-h, and 168-h). Data presented as mean ± SEM.

The average serum 25(OH)D concentration was 28.0 ± 2.5 ng/mL upon study enrollment (Figure 3). Upon enrollment, five subjects (36%) had a serum 25(OH)D concentration greater than 32 ng/mL. Of the nine participants (64%) with serum 25(OH)D concentrations less than 32 ng/mL, three (21%) subjects had a concentration less than 20 ng/mL, and one (7%) of which had a concentration less than 10 ng/mL.

Consistent with the seasonal regulation [75], serum 25(OH)D concentrations significantly (*p* < 0.05) decreased by approximately 10% from Bsl to Pre (Figure 2). Surprisingly, serum 25(OH)D concentrations significantly (*p* < 0.05) increased immediately after the intense exercise protocol. Within minutes, however, serum 25(OH)D concentrations subsequently decreased (*p* < 0.05) and leveled during the following days. 

### 3.3. Peak Isometric Force

**Figure 4:**
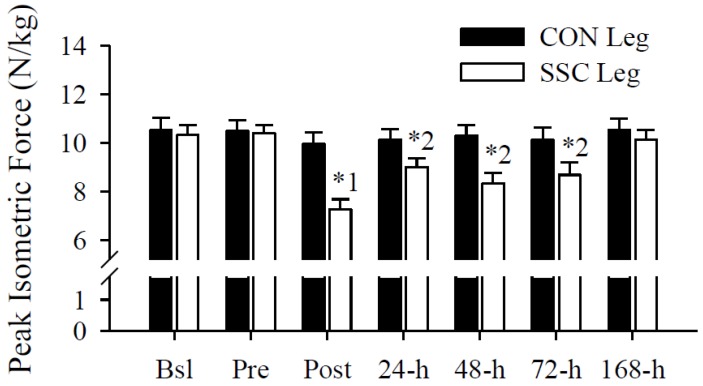
Peak isometric force (N/kg). Single leg peak isometric forces were significantly (leg, time interaction, *p* < 0.05) different following the SSC protocol. Single-leg peak isometric forces were not significantly different within the control (CON) leg across time, but within the SSC leg, decreased immediately (^1^ *p* < 0.05 *vs.* Bsl, Pre, 24-h, 72-h, and 168-h) and remained impaired several days (^2^ *p* < 0.05 *vs.* Bsl, Pre, and 168-h) after the intense exercise protocol. Peak isometric forces were also significantly (* *p* < 0.05) decreased in the SSC leg compared to those in the CON leg at Post, 24-h, 48-h, and 72-h. Data presented as mean ± SEM.

Peak isometric force significantly (*p* < 0.05) decreased immediately and remained significantly (*p* < 0.05) impaired several days after the exercise protocol (Figure 4). The immediate and persistent deficits in peak isometric force were apparent within the SSC leg across time (*p* < 0.05) and compared the corresponding CON leg (*p* < 0.05). At 168-h, peak isometric force returned to Pre and CON leg values.

### 3.4. Serum 25(OH)D Predicts Immediate and Persistent Muscular Weakness after a Muscle-Damaging Event

Pre serum 25(OH)D concentrations significantly (*p* < 0.05) predicted muscular weakness (CON *vs.* SSC Leg) immediately (*i.e.*, Post) but not 24-h after the intense exercise protocol (Table 1). Serum 25(OH)D concentrations at Pre significantly (*p* < 0.05) predicted muscular weakness (CON *vs.* SSC Leg) at 48-h and 72-h after the intense exercise protocol (Table 1).

In a sensitivity analysis, we repeated the multiple linear regression analyses (Table 1) regarding the prediction of muscular weakness following the intense exercise protocol. Excluding the Δ in Serum 25(OH)D from the multiple linear regression analyses did not change the significance of the models reported in Table 1. However, excluding the Δ in serum 25(OH)D increased the *p*-value from 0.04 to 0.08 for Pre serum 25(OH)D and decreased the model-statistical power from 86% to 59%. It is likely that the shift in significance and decrease in statistical power at 72-h after excluding the Δ in serum 25(OH)D from the multiple linear regression model is due to the size of our sample.

**Table 1:** Higher serum 25(OH)D concentrations predict a lower deficit ^1^ in muscle strength immediately (*i.e.*, Post) and days (*i.e.*, 48-h and 72-h) after intense exercise.

	Coefficient		
	Unstandardized (±SE)	Standardized	*p*
*Post*			
Constant	6.23 (±4.12)		
Pre serum 25(OH)D	−0.28 (±0.11)	−0.68	0.03
Δ serum 25(OH)D	−0.11 (±0.10)	−0.27	0.29
Age	−0.06 (±0.08)	−0.17	0.50
BMI	−0.28 (±0.13)	−0.57	0.06
*r*^2^ = 0.51, adjusted *r*^2^ = 0.30			
*24-h*			
Constant	2.41 (±4.00)		
Pre serum 25(OH)D	−0.17 (±0.10)	−0.52	0.14
Δ Serum 25(OH)D	−0.04 (±0.10)	−0.12	0.70
Age	−0.03 (±0.07)	−0.13	0.67
BMI	−0.04 (±0.13)	−0.10	0.77
*r*^2^ = 0.24, adjusted *r*^2^ = 0.00			
*48-h*			
Constant	7.24 (±4.43)		
Pre serum 25(OH)D	−0.28 (±0.12)	−0.68	0.04
Δ Serum 25(OH)D	−0.14 (±0.11)	−0.33	0.23
Age	−0.07 (±0.08)	−0.23	0.40
BMI	−0.10 (±0.11)	−0.29	0.33
*r*^2^ = 0.45, adjusted *r*^2^ = 0.21			
*72-h*			
Constant	6.40 (±3.34)		
Pre serum 25(OH)D	−0.21 (±0.09)	−0.63	0.04
Δ Serum 25(OH)D	−0.15 (±0.08)	−0.45	0.09
Age	−0.08 (±0.06)	−0.29	0.26
BMI	−0.10 (±0.11)	−0.26	0.36
*r*^2^ = 0.52, adjusted *r*^2^ = 0.31			

^1^ Peak isometric force difference between the CON and SSC legs at Post, 24-h, 48-h, and 72-h; Δ, Change in serum 25(OH)D from Bsl to Pre; BMI, Body mass index.

### 3.5. Plasma AST and ALT

**Figure 5:**
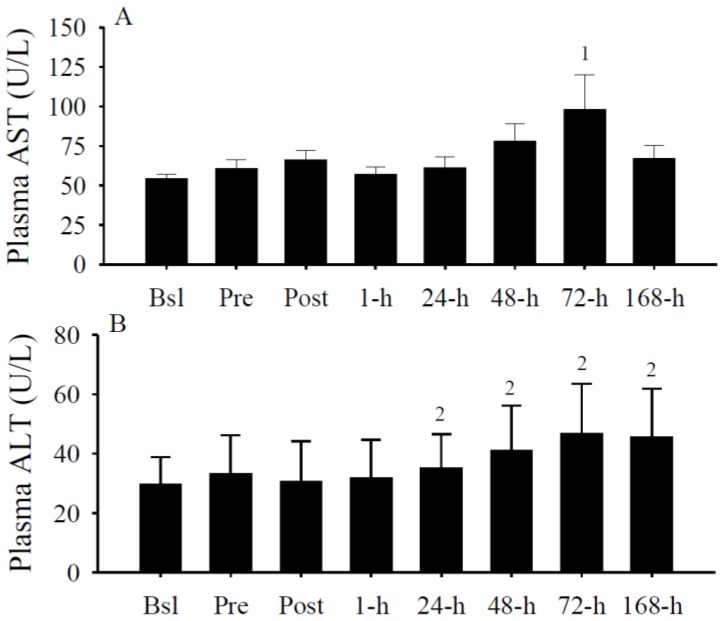
Plasma aspartate aminotransferase (AST) (U/L) and ALT (U/L) concentrations. (**A**) Plasma AST was significantly increased at 72-h (^1^ *p* < 0.05 *vs.* Bsl). (**B**) Plasma ALT was significantly (^2^ *p* < 0.05 *vs.* Post) increased at 24-h, 72-h, and 168-h. Data presented as mean ± SEM.

Increases in circulating AST and ALT are indices of skeletal muscle damage [76,77]. Days after the exercise protocol, plasma AST (Figure 5A) and ALT (Figure 5B) concentrations were significantly (both *p* < 0.05) increased, thereby imply the presence of skeletal muscle damage.

### 3.6. Serum Cytokine Concentrations

**Figure 6:**
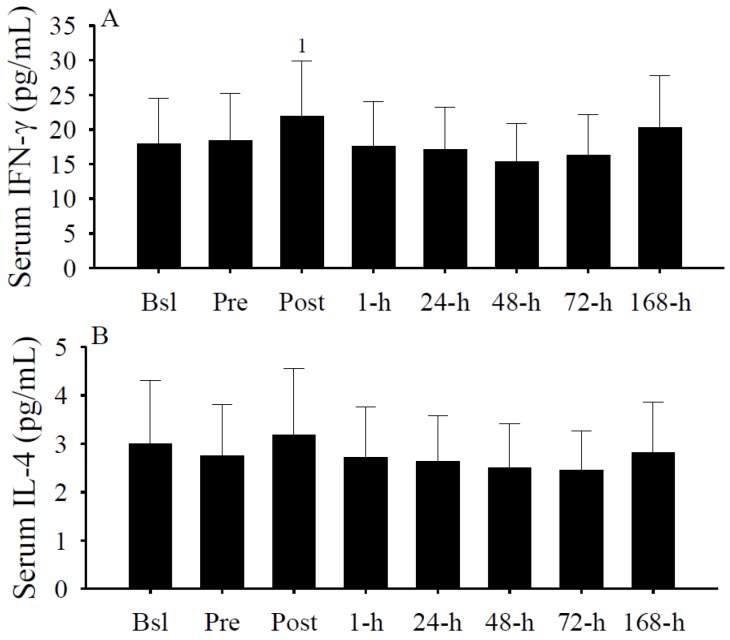
Serum IFN-γ and IL-4 concentrations (pg/mL). Serum IFN-γ concentrations (**A**) were significantly (^1^ *p* < 0.05 *vs.* 1-h, 48-h, and 72-h) increased at Post. Serum IL-4 concentrations (**B**) were not significantly different. Data presented as mean ± SEM.

Serum IFN-γ concentrations (Figure 6A) were significantly (*p* < 0.05) increased immediately after the exercise protocol. Although serum IL-4 concentrations were also elevated (Figure 6B), concentrations were not significantly different. Thus, IFN-γ was elevated while IL-4 was not significantly changed immediately after intense exercise.

### 3.7. Plasma Calcium, PTH, and Albumin Concentrations

**Figure 7:**
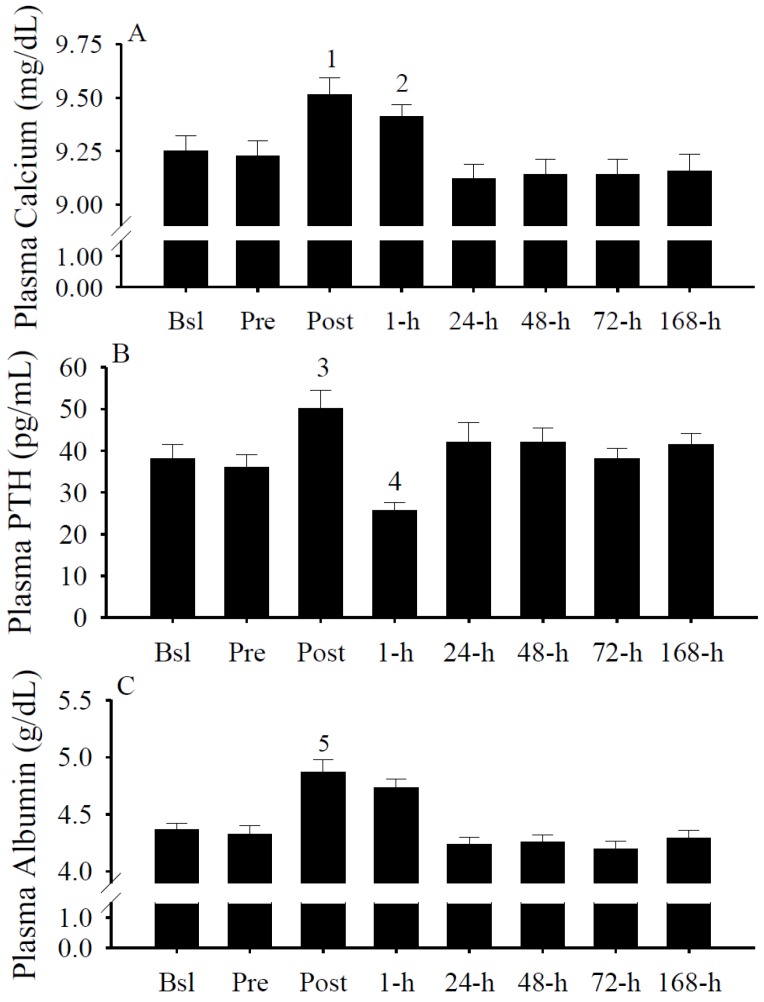
Plasma calcium (mg/dL), parathyroid hormone (PTH; pg/mL), and albumin (g/dL) concentrations. (**A**) Total plasma calcium concentrations were significantly increased at Post (^1^ *p* < 0.05 *vs.* Bsl, Pre, 24-h, 48-h, 72-h, and 168-h) and 1-h (^2^ *p* < 0.05 *vs.* 24-h, 48-h, 72-h, and 168-h). (**B**) Plasma PTH concentrations were significantly increased at Post (^3^ *p* < 0.05 *vs.* Bsl, Pre, 1-h, and 72-h) and significantly decreased at 1-h (^4^ *p* < 0.05 *vs.* 24-h, 48-h, and 168-h). (**C**) Plasma albumin concentrations were significantly increased at Post (^5^ *p* < 0.05 *vs.* Bsl, Pre, 1-h, 24-h, 48-h, 72-h, and 168-h). Data presented as mean ± SEM.

Immediately following the exercise protocol (*i.e.*, Post), plasma calcium (Figure 7A) and PTH (Figure 7B) concentrations were significantly (both *p* < 0.05) increased. At 1-h, calcium concentrations remained significantly (*p* < 0.05) increased and plasma PTH concentrations significantly (*p* < 0.05) decreased. Immediately following intense exercise, albumin concentrations significantly (*p* < 0.05) increased (Figure 7C).

### 3.8. IFN-γ and Albumin Predict Serum 25(OH)D Changes

Circulating IFN-γ increases predicted (*p* < 0.001) serum 25(OH)D decreases, while albumin increases predicted (*p* < 0.005) serum 25(OH)D increases (Table 2). 

**Table 2:** Circulating albumin increases predict serum 25(OH)D increases, while IFN-γ increases predict serum 25(OH)D decreases.

	Coefficient		
	Unstandardized (± SE)	Standardized	*p*
Constant	75.99 (±6.32)		
Δ IFN-γ	−0.60 (±0.09)	−0.60	<0.001
Δ iPTH	0.06 (±0.09)	0.06	0.48
Δ Calcium	19.7 (±10.6)	0.19	0.07
Δ Albumin	28.1 (±9.3)	0.32	<0.005
*r*^2^ = 0.36, adjusted *r*^2^ = 0.33			

Δ, change from Bsl.

### 3.9. Gender Differences

In the secondary analysis investigating the difference between genders, serum 25(OH)D, IFN-γ, IL-4, PTH, AST, ALT, peak isometric force, and the difference in peak isometric force between the CON and SSC legs were not significantly different between males and females (data not shown). However, circulating calcium (males, 9.33 ± 0.06 *vs.* females, 9.09 ± 0.08 mg/dL) and albumin (males, 4.48 ± 0.05 *vs.* females, 4.16 ± 0.07 g/dL) concentrations were significantly (both *p* < 0.05) different between genders.

## 4. Discussion

In the present investigation, we provide new data identifying that muscular weakness immediately (*i.e.*, Post) and several days (*i.e.*, 48-h and 72-h) after intense exercise were inversely predicted by pre-exercise serum 25(OH)D concentrations. Additionally, IFN-γ increases predicted serum 25(OH)D decreases, while albumin increases predicted serum 25(OH)D increases. These unique findings establish the importance of IFN-γ and albumin on the regulation of serum 25(OH)D, and that the serum 25(OH)D concentration prior to exercise could be a determinant of muscular strength after intense exercise.

The novel finding of the present investigation was the inverse association between pre-exercise serum 25(OH)D and both immediate and persistent muscular weakness after intense exercise. Pre-exercise serum 25(OH)D concentrations, however, did not predict muscular weakness 24-h after the intense exercise protocol, collectively suggesting a temporal sensitivity of skeletal muscle strength to vitamin D levels following muscular insult. 

Muscular weakness is a unique measure of muscle recovery because it spans the degenerative and regenerative events occurring at the molecular and cellular levels [44]. Furthermore, muscular weakness persists until repair is complete, suggesting that a faster recovery in skeletal muscle strength reflects a faster progression through the degenerative and regenerative events following muscular insult. In experimental rats, vitamin D treatment accelerated the recovery in muscular strength after injury [19]. Vitamin D treatment also increased serum 25(OH)D concentrations [19], which presumably, maintained substrate availability for the conversion of vitamin D to 1,25(OH)D and increased the expression (message and protein) of the vitamin D-receptor (VDR) [21]. Despite cell and experimental animal data supporting the therapeutic influence of vitamin D on the muscle recovery after muscular insult, results in human suggest otherwise.

Contrasting with the results here, Ring et al. [55] reported that vitamin D status was not influential on muscular weakness after exercise. The reason for the conflicting reports could relate to different experimental protocols, including the muscle(s) or muscle group(s) exercised, damaged and/or tested, disparate contraction modalities (*i.e.*, eccentric *vs.* eccentric-concentric contraction cycles), or the volume, duration and intensity of the exercise protocol. Additionally, the non-significant linear regression reported previously consisted of 2-d post isometric force and serum 25(OH)D concentration data [55]. Although we did not observe a significant difference 48-h after the intense exercise protocol, it is probable that serum 25(OH)D concentrations were modulated by intense-eccentric contractions [55], and consequentially, confounded the interpretation between vitamin D and muscular strength. To avoid the potential confounding influence of exercise in the present study, pre-exercise serum 25(OH)D concentrations were related to post-exercise muscle weakness. 

Consistent with the postulate that exercise modulates circulating vitamin D, we identify a transient 25(OH)D increase in the circulation immediately after intense exercise. The serum 25(OH)D concentration increases immediately after the intense exercise protocol was preceded by an initial (*i.e.*, Bsl to Pre) and followed by a second (*i.e.*, Post to 1-h) decrease. The initial decrease is consistent with data demonstrating the serum 25(OH)D concentration response during the winter [75]. The second decrease, however, suggests that serum 25(OH)D is temporally sensitive to intense exercise. Interestingly, cytokine and protein shifts in the circulation could contribute, in part, to the observed 25(OH)D fluctuations (see Table 2). 

Interferon-γ is an inflammatory cytokine that regulates vitamin D metabolism [60,61,78,79]. In immune and endothelial cells, IFN-γ impairs CYP27A1 message and protein expression [80], and thereby implying its potential role of impeding 25(OH)D production. Furthermore, IFN-γ accelerates the conversion of 25(OH)D to 1,25(OH)D [60,61,78,79] by increasing 1αOHase expression [62,64,65,81,82]. Supporting the influence of IFN-γ on vitamin D metabolism *in vivo*, we illustrated previously that IFN-γ tends to inversely correlate with 25(OH)D and significantly correlates (positive) with 1,25(OH)D [59], suggesting 25(OH)D decreases and 1,25(OH)D increases with increasing IFN-γ in the circulation. Based on the results here and those reported elsewhere, we propose that IFN-γ contributes to the circulating 25(OH)D decrease by impairing the production and accelerating the catabolism of 25(OH)D. However, confirmation of the proposed hypothesis requires further investigation.

Interleukin-4 is another inflammatory cytokine that has been implicated in the regulation of vitamin D metabolism. In human monocytes, IL-4 upregulated the message expression of CYP27B1 and the VDR [65]. Although message expression increased, IL-4 did not increase the activity of CYP27B1 [65]. Strikingly, IL-4 increased the activity of CYP24A1 and the conversion of 25(OH)D to 24,25(OH)D in monocytes [65]. These unique findings suggest that IL-4 could decrease 25(OH)D concentrations by accelerating catabolism. However, we were unable to detect a significant difference in IL-4 concentrations prior to or following the intense exercise protocol. Thus, based on our findings, it is unclear if IL-4 contributes to the fluctuating serum 25(OH)D concentrations observed during the winter and following intense exercise.

Another unique finding of the present investigation was the prediction of serum 25(OH)D concentrations by albumin. Albumin is a plasma protein synthesized in the liver that maintains colloid osmotic pressure [83]. Following short-term, high intensity exercise, albumin increases in the circulation and expands blood volume [84,85,86]. In the circulation, albumin binds to vitamin D metabolites [87]. The predication of increasing 25(OH)D by albumin is plausibly the product of increased hepatic secretion of albumin and its binding to vitamin D in the blood. However, additional research investigating the influence of albumin on vitamin D concentration in the circulation is required. 

Calcium and PTH regulate circulating 25(OH)D concentrations. Low calcium concentrations in the blood stimulate the parathyroid gland to secrete PTH. The increase in PTH stimulates the conversion of 25(OH)D to 1,25(OH)D [88,89,90] and increases the circulating calcium. Increases in dietary and serum calcium increase the catabolism of 25(OH)D to 24,25(OH)D [91]. In this investigation, we found an increase in plasma calcium concentrations immediately following the intense exercise protocol that was resolved by the following day (Figure 7A). PTH concentrations displayed a transient fluctuation after (*i.e.*, Post and 1-h) the intense exercise protocol (Figure 7B). Although vitamin D metabolism is regulated by calcium and PTH, neither the calcium or PTH predicted the deviation in serum 25(OH)D concentrations after intense exercise (Table 2).

In addition to those discussed above, there are limitations to this study that should be considered with future investigations. First, we did not obtain skeletal muscle biopsies to assess local damage, vitamin D metabolites, or the enzymes involved with vitamin D metabolism. Second, we did not control fluid or dietary intake prior to, during, or following intense exercise. Third, it is unknown if the results here can be extrapolated to other inflammatory modalities. Fourth, calcium and albumin concentrations were significantly different between genders. Calcium and albumin concentrations, however, were within the recommended clinical reference ranges (calcium, 8.4–10.4 mg/dL; albumin, 3.3–4.8 g/dL) for both genders, and therefore, it is unclear if this statistical difference is of physiological impact. Along these lines, serum 25(OH)D concentrations and peak isometric forces were not significantly different between male and female subjects as well. Next, this study consisted of 14 subject’s total. Future investigations are encouraged to include larger sample sizes when investigating the potential influence of vitamin D on skeletal muscle strength after injury. Finally, it is unknown skeletal muscle damage or fatigue-related factors, or both, were contributing to muscular weakness after the imposed exercise protocol. However, it is noteworthy that circulating biomarkers of skeletal muscle damage, and specifically AST and ALT, were elevated concurrently with deficits in skeletal muscle strength during the days following the intense exercise protocol. Thus, these results tend to support the possibility of the presence of skeletal muscle damage.

## 5. Conclusions and Implications

Muscle weakness hinders millions of people worldwide every year and is mediated by a variety of conditions, including aging, disease, inactivity, limb immobilization, repetitive use, and intense or unaccustomed exercise. In the present investigation, we reveal that muscular weakness after an intense exercise bout is abrogated with increasing serum 25(OH)D concentration prior to exercise. However, this was apparent immediately and several days (2-day and 3-day), but not 1-day, after intense exercise. Additionally, IFN-γ and albumin predicted serum 25(OH)D concentration fluctuations, and due on these fluctuations, we recommend caution when interpreting serum 25(OH)D concentrations immediately after intense exercise and/or during an acute inflammatory response. We conclude that maintaining an adequate serum 25(OH)D concentration could attenuate muscular weakness after intense exercise. Given the feasibility of increasing 25(OH)D concentrations in the blood, future research investigating the influence of diverse vitamin D interventions on the alleviation of muscular weakness after muscular insult are encouraged in humans. 
